# Pocket Therapeutics and Dose Book, with Classification and Explanation of the Actions of Medicines

**Published:** 1879-08

**Authors:** 


### BIBLIOGRAPHICAL.

Pocket Therapeutics and Dose Book, with Classification and Explanation of the Actions of Medicines. By Morse Stewart, Jr., B.A., M.D. Second Edition, Revised and Enlarged. Detroit, Mich. Geo. D. Stewart. 1878.

As the name indicates, this is truly a volume for the pocket. Maximum and minimum doses of medicines are given in troy, with their equivalents in metric weights. An index and definition of diseases and their remedies, and also of pharmaceutical names, with poisons and their antidotes will be found in the little book. The classification of medicines, found in the first part, occupies only eleven pages, and while the divisions are named from the therapeutic results obtained, the names of classes indicate the physiological action to be expected. For example, "hæmatics" are said to cause a change in the blood. Now, if the physiological actions of remedies are expressed at all in classification we must be content, since most authors, until very recently, gave no attention to this important point.

The book, for the purpose intended, will be found useful and convenient for practitioners and students.

